# Investigating sleep quality on an inpatient psychiatry ward

**DOI:** 10.1192/bjo.2021.525

**Published:** 2021-06-18

**Authors:** Joshua Hughes, Rhianne Thomas, Jonathan Macklin, Jonathan Owen

**Affiliations:** 1 NHS Dumfries and Galloway; 2 University of Edinburgh

## Abstract

**Aims:**

Sleep is essential for optimal physiological functioning, but often interrupted in hospital settings. Disturbed sleep is associated with relapse of mood disorders and multiple comorbidities including impaired immunological function and increased cardiovascular risk. There are unique environmental challenges on psychiatry wards, such as overnight monitoring. Recent studies highlight the importance of evaluating and managing inpatient sleep disturbance. Aims include exploring the extent to which patients’ sleep is impacted by inpatient admission, elucidating causes of sleep disturbance and determining ways to improve sleep during admission.

**Method:**

Patients aged 18–65 years, who consented and were expected to be inpatients for a week, were approached after 72 hours of admission (n = 35). Quantitative and qualitative data, including on pre-hospital and hospital sleep quantity and quality, were gathered, as part of a cohort characterisation. Questionnaires using Pittsburgh Sleep Quality Index elements were used to gather data. Offering earplugs as a sleep-aid intervention was implemented, with sleep quantity and quality reassessed 72 hours post-intervention. In response to feedback, sound monitoring at regular intervals overnight was undertaken using a decibel-metre to determine noise baseline and variation.

**Result:**

All patients approached agreed to participate. Pre-hospital average sleep quantity was 5.2 hours, with restedness score of 4.3, and 71% patients rating their sleep as ‘bad’. After 72 hours post-admission, average sleep length was 6.5 hours and restedness 5.3. Of patients who accepted earplugs (59%), there were improvements to mean sleep quality and quantity (7.6 hours), with 86% patients rating earplugs helpful. All patients surveyed thought that earplugs should be offered routinely on admission. 70% of patients were prescribed benzodiazepines or z-drugs as required. Self-reported factors affecting sleep included noise, psychiatric symptoms and medication side effects, with 13 patients mentioning the former. Sound monitoring recorded an average decibel level with a range of 35–75 dB, with peaks reaching 95 dB.

**Conclusion:**

Poor sleep in hospital is widespread. There is a need to understand and address modifiable environmental and ward factors implicated in sleep disturbance within inpatient settings. Pharmacological options for sedation are common, but it is important to focus on alternative options of low-cost, non-pharmacological interventions which promote sleep optimisation and enhance inpatient care.

